# Effects of Chronic PPAR-Agonist Treatment on Cardiac Structure and Function, Blood Pressure, and Kidney in Healthy Sprague-Dawley Rats

**DOI:** 10.1155/2009/237865

**Published:** 2009-06-11

**Authors:** Eileen R. Blasi, Jonathan Heyen, Michelle Hemkens, Aileen McHarg, Carolyn M. Ecelbarger, Swasti Tiwari

**Affiliations:** ^1^Safety Pharmacology, Pfizer, 10777 Science Center Drive CB4/2100, San Diego, CA 92121, USA; ^2^Department of Medicine, Georgetown University, 4000 Reservoir Rd. NW, Washington, DC, 20057, USA

## Abstract

PPAR-*γ* agonists have been associated with heart failure (HF) in diabetic patients. These incidences have been reported mostly in patient populations who were at high risk for HF or had pre-existing impaired cardiovascular function. However, whether there are similar effects of these agents in subjects with no or reduced cardiovascular pathophysiology is not clear. In this study, the effects of chronic treatment with PD168, a potent peroxisome proliferator activated receptor (PPAR) subtype-*γ* agonist with weak activity at PPAR-*α*, and rosiglitazone (RGZ), a less potent PPAR-*γ* agonist with no PPAR-*α* activity, were evaluated on the cardiovascular-renal system in healthy male Sprague-Dawley (SD) rats by serial echocardiography and radiotelemetry. Rats were treated with vehicle (VEH), PD168, @ 10 or 50 mg/kg·bw/day (PD-10 or PD-50, resp.) or RGZ @ 180 mg/kg·bw/day for 28 days (*n* = 10/group). Relative to VEH, RGZ, and both doses of PD168 resulted in a significant fall in blood pressure. Furthermore, RGZ and PD168 increased plasma volume (% increase from baseline) 18%, 22%, and 48% for RGZ, PD-10, and PD-50, respectively. PD168 and RGZ significantly increased urinary aldosterone excretion and heart-to-body weight ratio relative to VEH. In addition, PD168 significantly decreased (10–16%) cardiac ejection fraction (EF) and increased left ventricular area (LVA) in systole (s) and diastole (d) in PD-10 and -50 rats. RGZ significantly increased LVAd; however, it did not affect EF relative to VEH. In conclusion, chronic PPAR-*γ* therapy may predispose the cardiorenal system to a potential sequela of structural and/or functional changes that may be deleterious with regard to morbidity and mortality.

## 1. Introduction

Peroxisome proliferator-activated receptors (PPARs) are part of the nuclear receptor superfamily that includes at least three members, that is, alpha, beta, and gamma encoded by distinct genes. They have principal roles in metabolism, for example, tissue energy storage and utilization. PPAR alpha is expressed in tissues like brown adipose tissue, liver, kidney, intestine, heart, and skeletal muscle, with significant fatty acid and cholesterol catabolism. It is a key regulator of energy homeostasis and plays a major role in lipid metabolism and glucogenesis. PPAR-*γ* on the other hand is present at high levels in adipose tissue, gut, brain, vascular cells, immune cells, and retina. PPAR-*γ* plays a role in adipocyte differentiation, glucose metabolism, and lipid homeostasis, and participates in monocyte/macrophage differentiation. In the heart, the expression of PPAR-*γ* is relatively lower than PPAR-*α* [1, 2]. However, despite its lower expression, a prominent role for PPAR-*γ* in cardiac function, particularly in the left ventricle has been suggested [3]. Murine models have been used to determine the direct effects of PPAR receptors in the heart [2, 4]. Studies using cardiac-specific deletion revealed that loss of PPAR-*γ* from the heart resulted in mild cardiac hypertrophy [2]. However, in the same study, it was shown that treatment with rosiglitazone, a commonly utilized PPAR-*γ* agonist, also resulted in cardiac hypertrophy, in both wild type and cardiac-specific PPAR-*γ* knockout mice. This suggested that the presence of PPAR-*γ* may be essential for normal cardiac development, but that treatment with exogenous PPAR-*γ* agonists might also be detrimental [2]. 

PPAR-*γ* agonists are widely used for the treatment of type2 diabetes. These agents alone or in combination with PPAR-*α* agonists lead to adipogenesis, increased energy storage, and reduced insulin-resistance and hyperglycemia [5–9]. In addition, these agents have been shown to reduce inflammation and atherosclerosis and thereby prove beneficial for the treatment of cardiovascular diseases (CVD) [10–12]. However, treatment with PPAR-*γ* agonists are commonly associated with unwanted side effects including edema and weight gain. Recently, treatment with rosiglitazone (Avandia) has been associated with increased risk of myocardial infarction and possibly death from cardiovascular causes in specific patient populations, that is, those with underlying cardio-renal diseases, however these effects are still controversial [13–16]. Despite these serious side effects, these drugs are still one of the most effective treatments for type 2 diabetes, and have additional anti-inflammatory actions that are of benefit. However, some studies suggest detrimental side effects may outweigh the benefits of currently available PPAR-*γ* agonists, and further refinement of the chemical nature of these drugs, and possibly combination with diuretics is needed [17, 18].

Type 2 diabetes is commonly associated with congestive heart failure (CHF), impaired cardiac function and reduced cardiac reserve. This association is especially strong in the elderly or in later stages of diabetes [19, 20]. Treatment with PPAR-*γ* agonists has been shown to further exacerbate cardiac dysfunction and induce cardiac hypertrophy in these patients [10, 13, 14]. So far, these effects of PPAR-*γ* agonists have been demonstrated mainly in animal models of disease or in subjects at high-risk for myocardial infarction. High-risk components include impaired coronary flow reserve, even in the absence of obstructive epicardial coronary disease, myocardial fibrosis, and maladaptive myocardial energy metabolism. However, it is unclear whether PPAR-*γ* agonists affect the healthy heart. Furthermore, PPAR-*γ* therapy is more commonly associated with edema, even to a greater extent than with CHF. Thus, it is possible that PPAR-*γ* agonists may precipitate symptoms of CHF through plasma volume expansion. Therefore, in this report the effects of chronic PPAR-*γ* agonist treatment were examined in normal healthy young, male Sprague Dawley (SD) rats, with no risk factors for CHF. Previously, it has been shown that SD rats develop edema and reduced BP with short-term PPAR-*γ* treatment [21]. 

In the present study, the dose-dependent effects of chronic PPAR-*γ* agonist administration on blood pressure and cardiac structure and function in relation to the development of edema was evaluated in healthy SD rats. In this study we used two different agonists of PPAR-*γ* with different potencies for PPAR-*γ*; 1) PD-0344168 (PD168), which is a coagonist that exhibits much stronger affinity for PPAR-*γ* with an EC_50_ of 5 nM for PPAR-*γ* while also displaying very weak alpha activity at 70 nM [21], and 2) Rosiglitazone (RGZ) which has an EC_50_ of 200 nM for PPAR-*γ* and inactivity at PPAR-*α* [22]. Rats were treated for 28 days with either 10 or 50 mg/kg PD168 or 80 mg/kg RGZ and blood pressure as well as cardiac structure and function were evaluated over the time course of treatment. 

## 2. Material and Methods

### 2.1. Animals and Experimental Design, (Figure 1)

Forty male SD rats of ~275 g were obtained from Charles River Laboratories (Wilmington, Mass, USA). Animals were maintained at all times under protocols approved by the Pfizer (International Animal Care and Use Committee) IACUC. Rats were randomly assigned to four treatment groups (*n* = 10/group): vehicle, PD-10 (10 mg/kg·bw/d), PD-50 (50 mg/kg·bw/d), or rosiglitazone (RGZ, 80 mg/kg·bw/d). Baseline echocardiographic examinations were performed. The day following baseline echocardiography, telemetry devices were surgically implanted in the rats for continuous blood pressure monitoring (20-second reading, once every 15 minutes, 24 hours a day). Animals were dosed by gavage one time per day for 28 days with either vehicle (0.5% methylcellulose) or one of the two doses of PD168 in 0.5% methylcellulose. At the end of the study, animals were euthanized by conscious decapitation. For pathological examination, hearts were quickly excised, rinsed in sterile saline, blotted dry and weighed. Hearts were placed in 10% neutral buffered formalin (NBF).

### 2.2. Clinical Chemistry

Blood was collected into vacutainer tubes containing either K^+^-EDTA or heparin, centrifuged at 3000 rpm, and plasma collected and stored at −80°C until further analysis. Glucose and electrolytes were analyzed by routine clinical chemistry protocols on a Hitachi analyzer; hematocrit and hemoglobin were determined on the Advia 120 hematology analyzer (Bayer), and adiponectin was assayed by an enzyme-linked immunoassay (ELISA, mouse-rat adiponectin kit, B-Bridge International, Mountainview, Calif, USA). Twenty-four-hour urine was collected at the end of the study to measure urinary excretion of electrolytes (Na^+^ and K^+^) and total protein (Hitachi clinical chemistry analyzer). Aldosterone was assayed using standard procedures via radioimmunoassay (RIA) using ^125^I-labeled aldosterone (Diagnostic Products Corporation, Los Angles, Calif, USA).

### 2.3. Echocardiography

Serial echocardiographic examinations were performed on day 1, 3, 5, 7, 14, 21, and 28 of dosing using the Agilent SONOS 5500 echocardiographic system equipped with a 15-MHz linear-array transducer (Agilent, Andover). Images were captured from rats lightly anesthetized with 1-2% isoflurane (AErrane; Baxter, Inc., Deerfield, Mass, USA) lying on their left side with the transducer placed on the left hemithorax. Two-dimensional parasternal long and short-axis images of the left ventricle as well as two-dimensional targeted M-mode tracings were recorded from the parasternal short-axis view at the level of the papillary muscles at a sweep speed of 150 mm/s. Doppler flow velocities were taken at the level of the mitral valve in the apical 4-chamber view with the Doppler probe placed at the edge of the mitral leaflets. All measurements were performed according to the recommendations of the American Society for Echocardiography leading-edge method from three consecutive cardiac cycles. Measurements and calculations used were as follows: End diastolic (EDV) and systolic (ESV) volumes were calculated from LV systolic (LVAs) and diastolic (LVAd) areas. Ejection fraction (EF) was calculated from systolic and diastolic volumes with the following formula: EF = (EDV − ESV)/EDV × 100%. 

### 2.4. Blood Pressure (BP)

All animals were instrumented with PA-C40 radiotelemetry units (Data Sciences Inc., St. Paul, Minn, USA) for conscious, unrestricted BP measurements as previously described [21]. Briefly, animals were anesthetized with 3% isoflurane and a 5-cm midline incision was made through the skin and muscle layer of the abdominal wall exposing the peritoneal cavity. A 1.5-cm segment between the renal arteries and the bifurcation of the iliac arteries was exposed and the aorta was cannulated to insert the indwelling radiotelemetry probe-flow catheter. The transmitter was sewn into the muscle layer and the abdominal wall was closed. Postoperative care included treatment with 0.1 mg/kg, s.c. buphrenorphine and monitoring of the animals, which were placed on thermogenic heating pads during recovery from anesthesia until sternal recumbency and alertness were obtained.

### 2.5. Statistical Analysis

Data were presented as mean ± SEM. The means for VEH, PD-10, and PD-50 groups were compared using one-way analysis of variance (ANOVA) followed by Holm-Sidak multiple comparisons test, if significant. Values of *p* < 0.05 were used to show significance for the planned comparisons between the means. The means for VEH and RGZ groups were compared separately using unpaired *t*-test, values of *p* < 0.05 were considered significant.

## 3. Results

### 3.1. Effect of PD168, a PPAR *α*/*γ* Agonist 

#### 3.1.1. Serum Glucose and Plasma Adiponectin Levels with Chronic PD168 Treatment in Normal Rats

**Figure 2:**
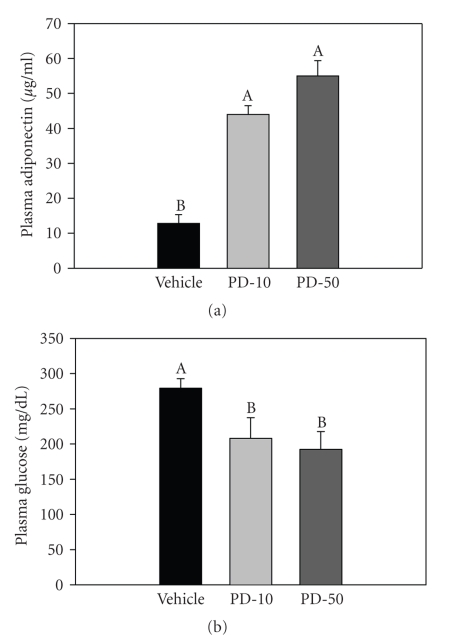
Plasma adiponectin (a) and serum glucose (b) levels in normal male SD rats chronically treated with vehicle (VEH), PD168 at a dose of 10 (PD-10) or 50 mg/kg·body weight (PD-50), (*n* = 10/treatment group). Means were compared using one-way ANOVA followed by the Holm-Sidak multiple comparisons test. Data were represented as means ± SEM. Bars designated with “A” are significantly greater in value than bars designated with “B” and so forth (by Holm-Sidak). *p* < 0.05 was considered significant.

After 4 weeks of treatment, plasma adiponectin levels were significantly higher in PD168-treated groups relative to the vehicle-treated group; 3.5 and 4.3 fold, for the 10 mg/kg and 50 mg/kg treatment groups, respectively (Figure 2(a)). Consistent with these changes, the serum glucose levels, under a fed state, were significantly lower (25% and 31% relative to vehicle for the 10 and 50 mg/kg treatment groups, resp.) after 4 weeks of treatments (Figure 2(b)). 

#### 3.1.2. Edema in Rats Treated with Chronic PD168

**Figure 3:**
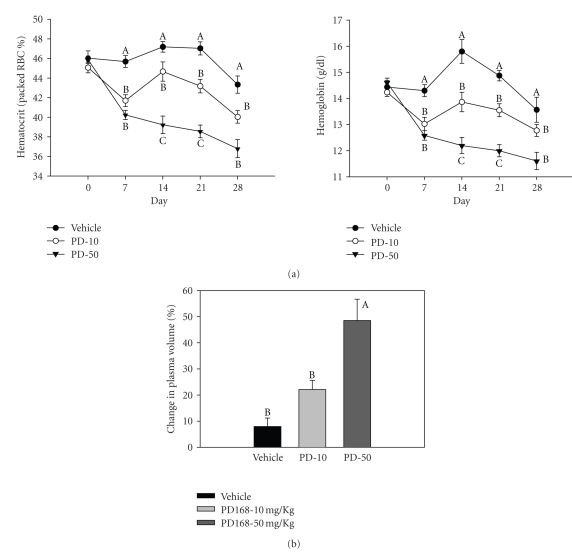
Hematocrit and hemoglobin levels in PD168-treated rats. (a) Percentage hematocrit (HCT) and hemoglobin levels and (b) percentage change in plasma volume, estimated indirectly by calculating percent change using baseline and final hemoglobin and hematocrit values in normal male SD rats chronically treated with vehicle (VEH), PD168 at the dose of 10 mg/kg·body weight (PD-10) or 50 mg/kg·body weight (PD-50), (*n* = 10/treatment group). Means were compared using one-way ANOVA followed by the Holm-Sidak multiple comparisons test. Data are represented as means ± SEM. Points designated with “A” are significantly greater in value than those designated with “B” (by Holm-Sidak). *p* < 0.05 was considered significant.

In all the blood collections after the start of treatments, that is, day 7, 14, 21, and 28, the hematocrit (HCT, % packed red blood cells), as well as the levels of hemoglobin (HBG) in the plasma of PD168-treated groups were significantly lower relative to the vehicle group (Figure 3(a)). At the end of the study, the total reductions in HCT were 6%, 10%, and 21% in vehicle, PD-10, and PD-50 groups, respectively. Similarly, the total reductions in plasma HBG were 8%, 11%, and 20% in vehicle, PD-10 and PD-50 groups, respectively. The change in plasma volume (%PV), as calculated using %HCT and HBG, was significantly higher in the PD-50 group and trended to be higher in PD-10 group, relative to vehicle-treated rats. The %PVs were 7.9 ± 3.4, 22.1 ± 3.5, and 48.5 ± 8.2 for vehicle, PD-10, and PD-50, respectively, (Figure 3(b)). 

In addition to HCT and HBG levels, gross observational signs of subcutaneous edema (swollen face, chest, extremities) coupled with interstitial fluid measured (from the thoracic cavity) were found in the PD168-treated rats. Four out of ten rats in PD 168-10 groups (40%) and seven out of ten rats in PD-50 group (70%) exhibited these signs. No such signs were observed in the vehicle-treated group (0%). Thoracic fluid retention in the PD-10 group ranged in volume from 0.2–0.5 ml, and in PD-50 group the fluid volume ranged from 0.6–1+ ml. Additionally, body weight gains were 59% and 62% respectively, for the PD-10 and PD-50 groups relative to 50% for the vehicle-treated group at the end of 28 days of treatment. Taken together, along with HCT and HBG values, edema and plasma volume expansion were evident with treatment.

#### 3.1.3. Evidence for an Activated Aldosterone Activity in Rats Treated with Chronic PD168

**Figure 4:**
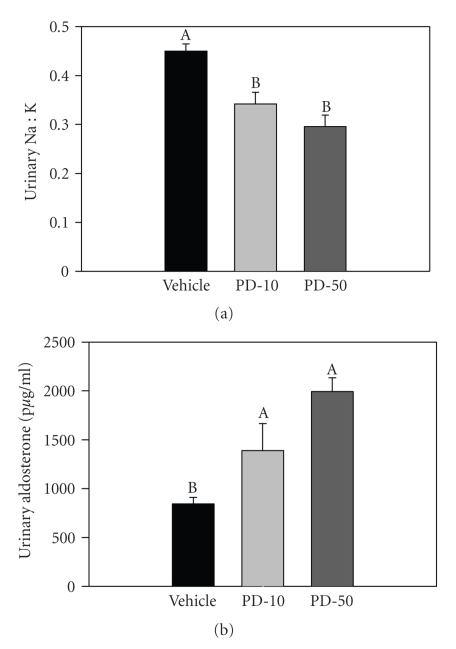
Aldosterone activity and excretion. (a) urinary sodium to potassium ratio (Na:K) and (b) aldosterone excretion in normal male SD rats chronically treated with vehicle (VEH), PD168 at the dose of 10 mg/kg·body weight (PD-10) or with PD168 at the dose of 50 mg/kg·body weight (PD-50), (*n* = 10/treatment group). Means were compared using one-way ANOVA followed by Holm-Sidak test. Data are represented as means ± SEM. Different letters represent significant differences between the groups with “A” being highest mean and so forth (*p* < 0.05 was considered significant).

Urine was collected for 24 hours before the end of the study to measure urinary excretion of sodium, potassium, and aldosterone. After 4 weeks of treatment, the urinary Na^+^/K^+^ ratio was found to be significantly decreased in PD168-treated rats (both 10 and 50 mg/kg) relative to vehicle-treated rats (Figure 4(a)). In addition, urinary aldosterone excretion was significantly increased by PD168 treatment (Figure 4(b)). Changes in these parameters indicate activation of aldosterone synthesis or reduced degradation or in PD168-treated rats relative to vehicle. 

#### 3.1.4. Cardiac-Hypertrophy by PD168 Administration

**Figure 5:**
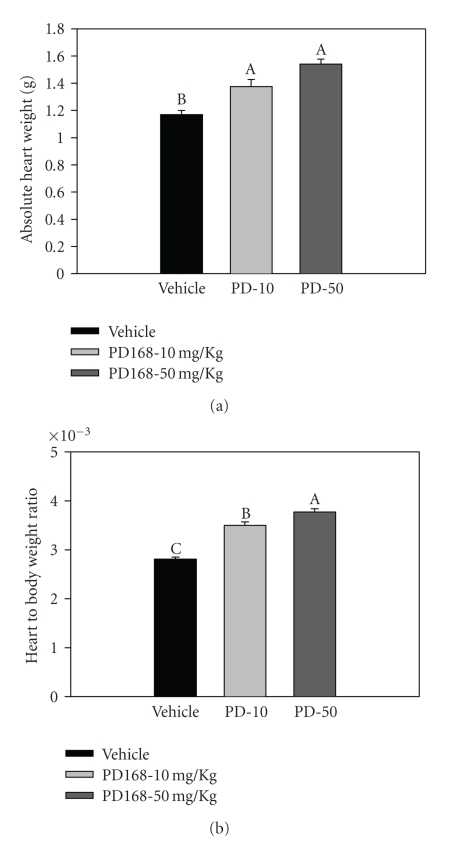
Cardiac hypertrophy in PD168 rats (a) absolute heart weight and (b) heart-to-body-weight ratio in normal male SD rats chronically treated with PD168 at the dose of 10 mg/kg·body weight (PD-10) or 50 mg/kg·body weight (PD-50) relative to vehicle (VEH), (*n* = 10/treatment group). Means were compared using one-way ANOVA followed by Holm-Sidak test. Data are represented as means ± SEM. Different letters represent significant differences between the groups with “A” being highest mean and so forth (*p* < 0.05 was considered significant).

Chronic PD168 treatment resulted in eccentric hypertrophy as evidenced by significantly increased absolute heart weight (Figure 5(a)). In addition, heart-to-body-weight ratios were also significantly increased by PD168 treatment (Figure 5(b)). Heart-to-body-weight ratios were 23.4% and 30.4% higher in the PD-10 and −50 groups, as compared to vehicle. However, compound-related morphologic changes such as signs of cardiac lesions, necrosis or fibrosis, were not observed in this study. 

#### 3.1.5. Left Ventricular Dysfunction by Chronic PD168 Treatment

**Figure 6:**
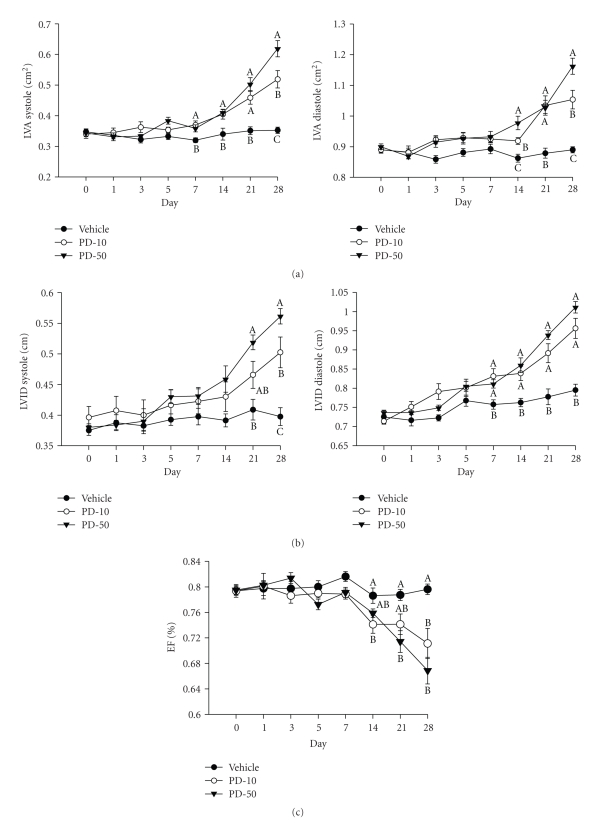
Left ventricular expansion and ejection fraction in PD168 treated rats. (a) Left ventricular area (LVA) in systole and diastole; (b) Left ventricular internal dimension (LVID) in systole and diastole in normal male SD rats chronically treated with vehicle (VEH), PD168 at the dose of 10 mg/kg·body weight (PD-10) or 50 mg/kg·body weight (PD-50), (*n* = 10/treatment group). Means were compared using one-way ANOVA followed by Holm-Sidak test. Data are represented as means ± SEM. Different letters represent significant differences between the groups with “A” being highest mean and “AB” not different from “A” or “B” and so forth (*p* < 0.05 was considered significant).

Echocardiography was performed a day before the start of the treatment (day 0) then every other day for one week (days 1, 3, 5, and 7); and thereafter, once a week until the end of the experiment (days 14, 21, and 28). Echocardiographic analysis revealed significant systolic and diastolic dysfunction as evidenced by an increase in the LVA in systole and diastole (Figure 6(a)). In systole, PD-10 and −50 resulted in a 53% and 80% increase in area, respectively, from baseline after 4 weeks. In diastole, PD-10 and − 50 resulted in an 18%, and 29% increase respectively, from baseline after 4 weeks. Figure 6(b) shows a significant increase in the left ventricular internal dimension (LVID) in PD168-treated groups over time, in both diastole and systole. Furthermore, ejection fraction was significantly decreased in both doses after day 14 of PD168 treatment (Figure 6(c)). By the end of 4 weeks of treatment, EF was decreased from baseline by 10% and 16% in PD-10 and −50, respectively. However, EF in the vehicle-treated group remained unchanged over the 4 week study.

#### 3.1.6. Effect of Chronic PD168 Treatment on BP and Total Protein Excretion

**Figure 7:**
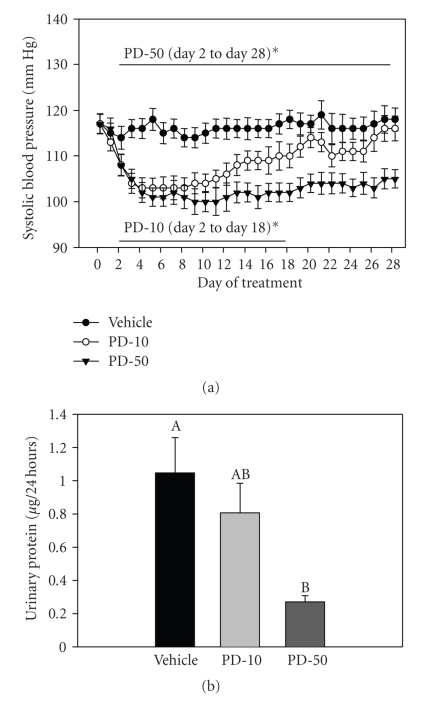
(a) Systolic blood pressure and (b) Urinary protein excretion in normal male SD rats chronically treated with vehicle (VEH), PD168 at the dose of 10 mg/kg·body weight (PD-10) or 50 mg/kg·body weight (PD-50), (*n* = 10/treatment group). Means were compared using one-way ANOVA followed by Holm-Sidak test. Data are represented as means ± SEM. Different letters represent significant differences between the groups with “A” being highest mean and “AB” not different from “A” or “B” and so forth (*p* < 0.05 was considered significant).

BP and heart rate (HR) were measured throughout the study using radiotelemetry. Systolic BP (SBP) was significantly reduced by both doses of PD168 (Figure 7(a)). At the lower dose (PD-10), SBP was significantly reduced relative to the vehicle group through day 17 of the treatment; however, it rebounded on day 18 and was no longer significantly different from vehicle for the remainder of the study. However, at the higher dose (PD-50), SBP remained significantly reduced by about 11% relative to the vehicle group, throughout the course of the 28 days of treatment. Diastolic BP showed the same trend as SBP (data not shown). There was a significant elevation in HR with PD-50 in the first 5 days, likely in response to the fall in BP. Total urinary protein excretion was measured to assess integrity of the glomerulus and associated filtration membranes. Protein excretion was found to be significantly lower in both PD168-treated groups related to vehicle (Figure 7(b)).

### 3.2. Effects of Rosiglitazone, a PPAR*γ* Agonist 

#### 3.2.1. Plasma Adiponectin and Serum Glucose Levels with Chronic Rosiglitazone Treatment in Normal Rats

**Figure 8:**
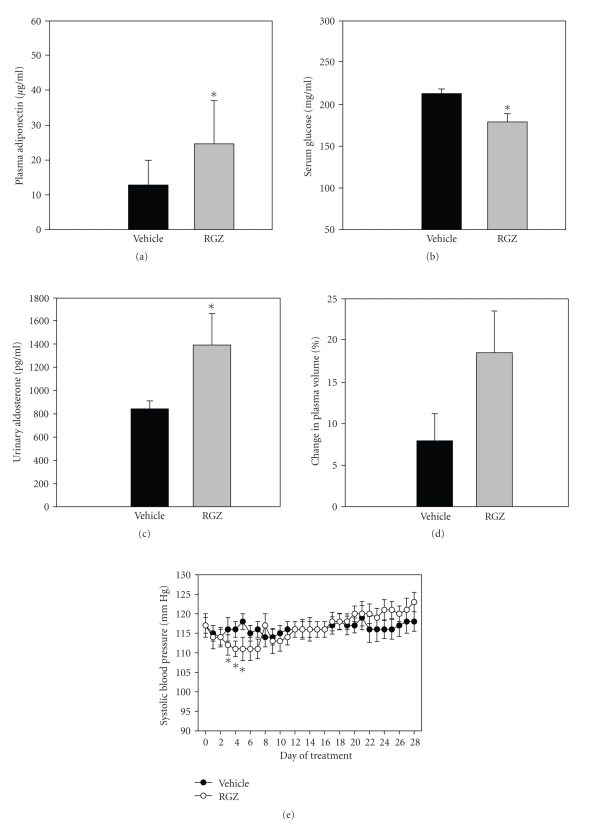
(a) Plasma adiponectin; (b) serum glucose; (c) Urinary aldosterone levels; (d) % Change in Plasma volume; and (e) Systolic blood pressure in normal male SD rats chronically treated with vehicle (VEH) or rosiglitazone at the dose of 80 mg/kg·body weight (RGZ), (*n* = 10/treatment group). Data were represented as means ± SEM. Means were compared using unpaired *t*-test. *indicates a significant difference between the groups (*p* < 0.05).

RGZ treatment significantly increased plasma adiponectin levels relative to the vehicle-treated group (Figure 8(a)) after 4 weeks. Consistently, serum glucose levels were significantly lower in RGZ treated groups relative to vehicle (Figure 8(b)).

#### 3.2.2. Urinary Aldosterone Excretion, Plasma Volume and Systolic Blood Pressure with Chronic Rosiglitazone Treatment in Normal Rats

Rats treated with RGZ had significantly increased urinary aldosterone levels relative to vehicle group (Figure 8(c)) in their 24 hour urine samples collected towards the end of the study. 

Percentage change in plasma volume (%PV) was calculated using %HCT and HBG levels, measured in the blood collected at baseline and at the end of the study. Percentage PV was trending higher in RGZ treated rats relative to vehicle (Figure 8(d); not significant). Furthermore, RGZ treated rats had a significant fall in systolic blood pressure (SBP) at treatment days 4 to 6 relative to vehicle (Figure 8(e)). After day 6, the SBP rebounded back and was no longer significantly different from vehicle for the remainder of the study.

#### 3.2.3. Effect of Chronic Rosiglitazone on Cardiac Structure and Function

**Figure 9:**
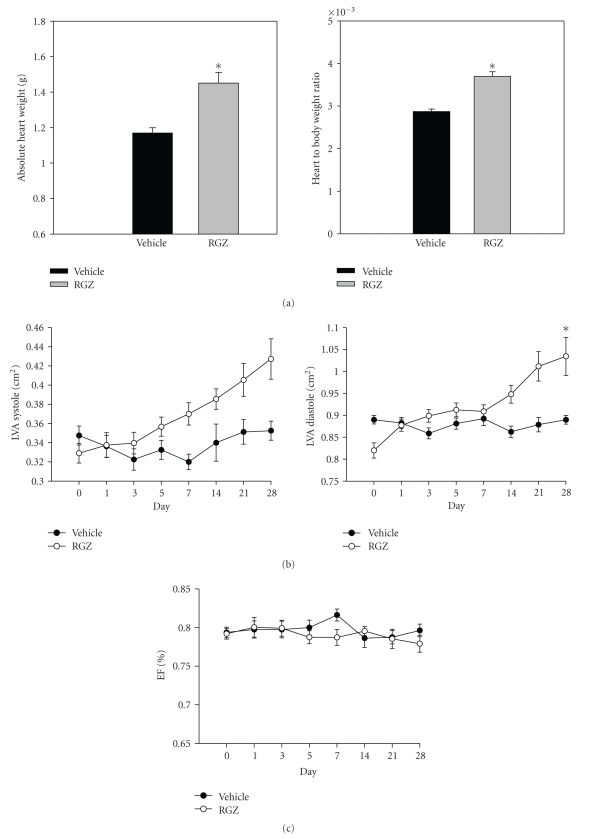
(a) Absolute heart weight and heart-to-body-weight ratio; (b) Left ventricular area (LVA) in systole and diastole; and (c) Ejection fraction (EFA) in normal male SD rats chronically treated with vehicle (VEH) or rosiglitazone at the dose of 80 mg/kg·body weight (RGZ), (*n* = 10/treatment group). Data were represented as means ± SEM. Means were compared using unpaired *t*-test. *indicates a significant difference between the groups (*p* < 0.05).

RGZ treatment resulted in cardiac hypertrophy as indicated by a significant increase in absolute heart weight as well as heart-to-body-weight ratio relative to the vehicle-treated group (Figure 9(a)). Furthermore, echocardiographic analysis revealed that RGZ resulted in a significant increase (21.5% from baseline) in the left ventricular area (LVA) in diastole relative to vehicle group after 4 weeks (Figure 9(b)). For LVA in systole there was a trend for an increase relative to vehicle group (9.8% from baseline), however, it was not significant. Furthermore, RGZ did not have any significant effect on the ejection fraction relative to the vehicle group (Figure 9(c)).

## 4. Discussion

The following study demonstrated the effects of chronic treatment with PD168 or RGZ on the hearts of healthy, young SD rats. PD168 has highly potent PPAR-*γ* activity (5 nM) with weaker PPAR-*α* activity (70 nM). RGZ, on the other hand has an EC_50_ of 200 nM for PPAR-*γ* and inactivity at PPAR-*α*. Chronic treatment with PD168 for 28 days resulted in reduced blood pressure and a fall in hematocrit early on followed by cardiac hypertrophy, expansion of the left ventricular area, and reduced ejection fraction. In addition, chronic PD168 treatment resulted in increased urinary aldosterone. The above parameters were modified, in general, in a dose-responsive fashion. The RGZ treatment for 28 days had a similar effect on aldosterone excretion and cardiac structure; however it did not increase plasma volume or reduce ejection fraction or blood pressure to the same extent of PD168. Overall, RGZ at this dose appeared to have less detrimental effects than PD168 which we believe could be due to the difference in the potency of the two drugs used at these doses. 

Plasma concentrations of PD168 were determined using a sensitive, drug-specific, HPLC-tandem mass spectrometry assay. The average steady state concentration was 460 ng/mL and 3300 ng/mL for PD168-10 mg and PD168-50 mg, respectively. The dose selection rationale for this study was aimed to achieve doses ranging from 0.5–10+ fold over the predicted human efficacious concentration (C-eff) in Zucker diabetic fatty rats that lowered glucose to below the target concentration of 200 mg/dL. The values above were from approximately 1 and 10-fold over the predicted human C-eff of 465 ng/mL. The pharmacokinetic assay measures total drug concentration over time, and at the doses evaluated in the study and the margins achieved we would consider this to be within the “proposed” clinical range (although this compound was discontinued and never tested in humans). As such, we would speculate that achieved highly potent PPAR-*γ* activity with very low alpha activity. However, at the higher dose we cannot rule out the potential effect of some alpha activity as it could not be directly measured.

### 4.1. Chronic PD168 Treatment Induced a Fall in Blood Pressure and Plasma Volume Expansion in Normal Rats

In agreement with findings from our previous study with shorter term PD-168 treatment [21], in the present study a significant fall in hematocrit (%) with PD168 (Figure 3(a)) was demonstrated. In addition, visible signs of subcutaneous edema coupled with interstitial fluid accumulation, weight gain and increases in plasma volume, measures of edema and volume retention, were found with PD168 treatment. There are numerous theories regarding how PPAR-*γ* agonists induce edema. One hypothesis is that this class of agents increase capillary permeability. In this regard, RGZ has been shown to modulate vascular permeability in a tissue-specific manner [18, 23–26]. Furthermore, the fall in blood pressure induced by PD-168 may also be a contributing factor to the edema since activation of aldosterone activity may facilitate increased salt and water retention through positive feedback mechanisms. In addition, the decrease in blood pressure preceded the edema after PD168 treatment. Our earlier studies also supported this finding in that a significant fall in blood pressure from 12 hours postdose was followed by a significant fall in hematocrit, initiated from day 3 of treatment [21].

An additional contributing mechanism for the edema may be PPAR-*γ* activation directly modulating distal sodium transport and inducing water retention. In this regard, it has been shown that thiazolidinedione-induced edema was partially blocked by antagonizing ENaC [27]. Mice with targeted knockout of the PPAR-*γ* receptor in the collecting duct (CD) of the kidney had less severe thiazolidinedione-(TZD) induced edema and also TZDs induced NaCl reabsorption [28]. Thus increased sodium reabsorption by chronic PD168 treatment found in the present study could be a direct effect of PPAR-*γ* on the renal distal tubule in these rats. Previously it has been shown that short-term rosiglitazone treatment reduces BP and increases sodium retention in healthy rats [29]. Furthermore, results from our previous study with shorter-term PD168 treatment have suggested initial activating changes in distal sodium and water transporter protein, ENaC and AQP-2 protein expression followed by their compensatory downregulation by day 5 [21]. As such, there is a possibility that PD168 increases sodium reabsorption by having a direct action on the renal salt and water proteins. However, whether PPAR-*γ* agonists directly increases the activity of ENaC is still controversial. Hong et al. [30] have found increased cell surface expression of ENaC by PPAR-*γ* agonists. While, Nofziger et al. [31] did not report any increase in PPAR-*γ*-induced Na+ influx via ENaC. Thus further studies are warranted to further elucidate the mechanism for TZD-associated edema. 

Regarding the mechanism for the fall in blood pressure, PPAR-*γ* agonists may have a direct vasodilatory action on the vasculature. In agreement with this hypothesis, rosiglitazone has been shown to cause vasorelaxation [32]. Specifically, Eto and colleagues have demonstrated that rosiglitazone causes attenuation of inward calcium currents and enhanced calcium-activated potassium currents in isolated vascular smooth muscle cells [33]. Modulation of calcium and potassium may be a possible mechanism for rosiglitazone-induced vasorelaxation since attenuated inward calcium currents and enhanced calcium-activated potassium currents would cause cellular hyperpolarization and, therefore, relaxation of the vessel. 

Previously, we reported that short term (1–5 days) treatment with PD168 (10 mg/kg) led to a significant fall in hematocrit and reduction in blood pressure. In the present chronic study, 28 days of repeat dosing showed similar effects. However, there was an attempt at physiological adaptation with regard to the fall in blood pressure which was attenuated after day 18th of the low-dose treatment (Figure 7(a)). These results may indicate some sort of compensatory mechanism in play. Furthermore, this compensation appears to be lost with the higher dose of this drug. Increased aldosterone in these rats may be playing a role in this inability to compensate. 

With regard to kidney, the PD-168-induced depressor effect may have a protective effect as suggested by significantly lower total protein excretion in the PD168-treated rats. This is in agreement with other studies which have demonstrated reduced urinary albumin excretion and renoprotection by thiazolidinediones [34–36]. The mechanisms for reduced protein excretion and renoprotection may be direct or indirect, due to the lowering of glucose levels or decreased blood pressure [35, 37–41].

### 4.2. Cardiac Hypertrophy and Reduced Cardiac Function

Cardiac hypertrophy in response to PPAR-*γ* exposure might be the result of direct or indirect actions of these agents. With regard to indirect actions, it is plausible that the cardiac hypertrophy found in our rats might reasonably be the result of volume overload [42, 43]. A similar observation was made by Arakawa and colleagues in diabetic SD rats treated with T-174, a drug with potent PPAR-*γ* agonistic activity and oral antidiabetic action [44]. The authors suggested that plasma-volume expansion led to cardiac volume overload, which then contributed to hypertrophy of the heart. This hypothesis is also supported by an in vitro study in cardiomyocytes by Sandra and colleagues [45] which demonstrated that cardiac hypertrophy associated with the treatment of PPAR-*γ* agonists was not due to the direct cardiac effects of PPAR agonists, for example, activation of cardiac PPAR-*γ* receptors or increased myocardial insulin sensitivity. Thus induction of cardiac hypertrophy could be an indirect rather than direct cardiomyocyte-specific effect of these agents. In this regard, rosiglitazone has been shown to induce cardiac hypertrophy in mice with cardiac-specific knockout of the PPAR-*γ* receptor, suggesting that this drug has some additional indirect effects to induce hypertrophy [2]. In the present study, plasma-volume expansion induced cardiac volume overload could be one such indirect mechanism for cardiac remodeling in PD168-treated rats. This is supported by the observation that the fall in hematocrit presented from day 7 onward, preceded changes in cardiac structure or function as assessed by echocardiography. Furthermore our previous short-term study with PD-168 at 10 mg/kg in SD rats clearly demonstrated that the fall in hematocrit started from day 1 of the treatment [21]. Furthermore, activation of the renin-angiotensin-aldosterone system (RAAS), as suggested by increased aldosterone excretion by PPAR-*γ* agonists could also contribute to hypertrophy and remodeling of the heart [46–48].

Nevertheless, there are some studies which support a direct effect of PPAR-*γ* agonists via PPAR-*γ* receptors on the heart producing detrimental effects on cardiac structure and function. In this regard, over-expression of PPAR-*γ* in the heart resulted in cardiac dysfunction [49]. Thus, cardiac dysfunction as indicated by reduced ejection fraction and left ventricular hypertrophy in our study may be the result of cardiac-specific effects of these agents. In contrast, there are some studies which suggest a protective effect of PPAR-*γ* agonists with regard to cardiac hypertrophy and heart function [50–52]. Despite data from various studies in diseased or non-diseased models, it is still controversial whether agonists of PPAR-*γ* have beneficial or deleterious effects on the heart.

Higher circulating levels of adiponectin are related to a reduced risk of cardiovascular disease in healthy individuals [53, 54]. Our finding of elevated plasma adiponectin along with cardiac dysfunction in PD168 treated rats counters these anti-inflammatory and anti-atherogenic properties of adiponectin. However, we do not believe that higher adiponectin level has any causative role in cardiac dysfunction found in this study. We believe that elevated plasma adiponectin levels and cardiac dysfunction are two independent effects of PPAR-*γ* agonists observed in our study. Nonetheless, some epidemiologic studies have reported that high adiponectin levels were associated with increased cardiovascular mortality in patients with chronic kidney disease, chronic heart failure and hemodialysis patients with type 2 diabetes mellitus [55–57]. Furthermore, it has been suggested that hypervolemic conditions in patient populations may modulate the effect of plasma adiponectin levels [57]. Thus, it is possible that the cardioprotective action of elevated adiponectin levels is disrupted by increased plasma volume found in PD168 treated rats in our study. 

In summary, results from the present study suggest that chronic treatment with a potent PPAR-*γ* agonist may introduce a risk factor for a sequel of events that may precipitate the eventual development of left ventricular dysfunction and CHF even in an otherwise healthy heart. Edema coupled with eccentric hypertrophy suggests volume overload to be one possible mechanism. Like normal rats in our study, most patients with no cardiovascular impairment can tolerate modest increases in intravascular volume; however, patients with impaired cardiovascular reserve or risk factors for cardiac dysfunction may manifest CHF signs and symptoms in the setting of this magnitude of volume expansion.

## Figures and Tables

**Figure 1:**
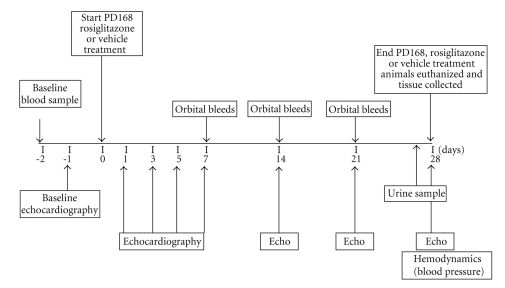
Schematic diagram of study design; echocardiography, echo.
